# An observer-blinded, cluster randomised trial of a typhoid conjugate vaccine in an urban South Indian cohort

**DOI:** 10.1186/s13063-023-07555-y

**Published:** 2023-08-03

**Authors:** Nikhil Sahai, Dilesh Kumar Arunachalam, Tim Morris, Andrew Copas, Prasanna Samuel, Venkata Raghava Mohan, Vinod Abraham, Joshua Anish Selwyn, Praveen Kumar, Winsley Rose, Veeraraghavan Balaji, Gagandeep Kang, Jacob John

**Affiliations:** 1 Wellcome Trust Research Laboratory, Department of G.I. Sciences, Christian Medical College Vellore, Vellore, India; 2 MRC Clinical Trials Unit, University College London, London, UK; 3 Department of Biostatistics, Christian Medical College Vellore, Vellore, India; 4 Department of Community Health, Christian Medical College Vellore, Vellore, India; 5 Department of Paediatrics, Christian Medical College Vellore, Vellore, India; 6 Department of Clinical Microbiology, Christian Medical College Vellore, Vellore, India

**Keywords:** Typhoid, Vaccine, TCV, Typhoid conjugate vaccine, Cluster randomised, Clinical trial, India

## Abstract

**Background:**

Typhoid fever causes nearly 110,000 deaths among 9.24 million cases globally and disproportionately affects developing countries. As a control measure in such regions, typhoid conjugate vaccines (TCVs) are recommended by the World Health Organization (WHO). We present here the protocol of a cluster randomised vaccine trial to assess the impact of introducing TyphiBEV® vaccine to those between 1 and 30 years of age in a high-burden setting.

**Methods:**

The primary objective is to determine the relative and absolute rate reduction of symptomatic, blood-culture-confirmed *S.* Typhi infection among participants vaccinated with TyphiBEV® in vaccine clusters compared with the unvaccinated participants in non-vaccine clusters. The study population is residents of 30 wards of Vellore (a South Indian city) with participants between the ages of 1 and 30 years who provide informed consent. The wards will be divided into 60 contiguous clusters and 30 will be randomly selected for its participants to receive TyphiBEV® at the start of the study. No placebo/control is planned for the non-intervention clusters, which will receive the vaccine at the end of the trial. Participants will not be blinded to their intervention. Episodes of typhoid fever among participants will be captured via stimulated, passive fever surveillance in the area for 2 years after vaccination, which will include the most utilised healthcare facilities. Observers blinded to the participants’ intervention statuses will record illness details. Relative and absolute rate reductions will be calculated at the end of this surveillance and used to estimate vaccine effectiveness.

**Discussion:**

The results from our trial will allow countries to make better-informed decisions regarding the TCV that they will roll-out and may improve the global supplies and affordability of the vaccines.

**Trial registration:**

Clinical Trials Registry of India (CTRI) CTRI/2022/03/041314. Prospectively registered on 23 March 2022 (https://ctri.nic.in/Clinicaltrials/pmaindet2.php?trialid=62548&EncHid=&userName=vellore%20typhoid). CTRI collects the full WHO Trial Registration Data Set.

**Supplementary Information:**

The online version contains supplementary material available at 10.1186/s13063-023-07555-y.

## Background

Globally, typhoid fever remains a major cause of high morbidity and mortality, with an estimated 9.24 million cases and 110,000 deaths being associated with the disease in 2019 [1]. It particularly affects developing nations in the sub-Saharan and South Asian regions where a majority of cases and deaths occur [1, 2]. More recent data from surveillance networks such as the Surveillance for Enteric Fever in Asia Project [3] and The Surveillance for Enteric Fever in India (SEFI) [4] show that the region continues to have a high incidence of Enteric fever (> 100 per 100,000 person-years). Management of this disease is hence a priority in South Asia, especially with the outbreaks of extremely drug-resistant *S.* Typhi in Pakistan [5] which threatens to spill over to its neighbouring countries.

The rise in drug-resistant cases of enteric fever limits treatment options and necessitates the implementation of other control measures such as improved sanitation and clean water supplies. However, the development of such infrastructure is a long-term solution, and the WHO recommends the programmatic of typhoid conjugate vaccines (TCVs) to reduce the burden of this disease [6] in the interim period. Currently, two TCVs are prequalified by the WHO—Typbar and TyphiBEV®. The former has been tested for its efficacy in phase III trials and is in programmatic use in multiple countries [6]. While the latter vaccine has been shown to produce immunogenicity similar to Typbar in a phase II trial [7], it lacks efficacy and impact data which hinders its uptake by Gavi-supported countries and limits the supply of TCVs worldwide.

Within India, data from the SEFI network and other studies have shown that TCV rollout is a cost-effective strategy to reduce the burden of typhoid in the country, and especially among children and young adults in urban areas who are disproportionally affected by it [2, 8, 9]. Consequently, the Ministry of Health in India may be receptive to TCV impact studies within the country, which could further lead to their implementation across high-burden settings. To promote the uptake of such vaccines and improve their supply in the country and worldwide, we plan to conduct a clinical trial to estimate the protective impact of the TyphiBEV® vaccine in Vellore, a South Indian city that has been shown to have a high burden of typhoid among children (1173 per 100,000 child-years of observation) [4]. Here, we describe the protocol we will use for this trial, which follows the SPIRIT guidelines [10]. A corresponding checklist can be found in Additional file 1.

## Aims

To determine the relative and absolute rate reduction of symptomatic, blood culture-confirmed *S.* Typhi infection among participants vaccinated with TyphiBEV® in vaccine clusters *vs*. the unvaccinated participants in non-vaccine clusters.

## Objectives

### Primary

1. To estimate the relative and absolute rate reduction (derived from the same model) of symptomatic culture-confirmed *S.* Typhi infection in TyphiBEV®-vaccinated participants in vaccine clusters compared to unvaccinated participants in non-vaccine clusters (total effect)

### Secondary

1. To estimate the relative and absolute rate reduction (derived from the same model) of symptomatic culture-confirmed S. Typhi infection among participants in vaccine clusters compared to participants in non-vaccine clusters (overall effect)
2. To estimate the relative and absolute rate reduction of symptomatic culture-confirmed *S.* Typhi infection in TyphiBEV®-vaccinated participants 1–14 years of age in vaccine clusters compared to unvaccinated participants 1–14 years of age in non-vaccine clusters
3. To estimate the relative and absolute rate reduction of febrile illness resulting in healthcare visits in vaccinated participants in vaccine clusters compared to unvaccinated participants in non-vaccine clusters
4. To describe safety outcomes associated with TyphiBEV® vaccination within the study population

### Exploratory

1. To estimate the relative and absolute rate reduction of symptomatic culture-confirmed S. Typhi infection in TyphiBEV® unvaccinated participants in vaccine clusters compared to unvaccinated participants in non-vaccine clusters (indirect effect)
2. To estimate the relative and absolute rate reduction of the incidence of hospitalised culture-confirmed S. Typhi infection in TyphiBEV®-vaccinated participants in vaccine clusters compared to unvaccinated participants in non-vaccine clusters

## Design

**Fig. 1:**
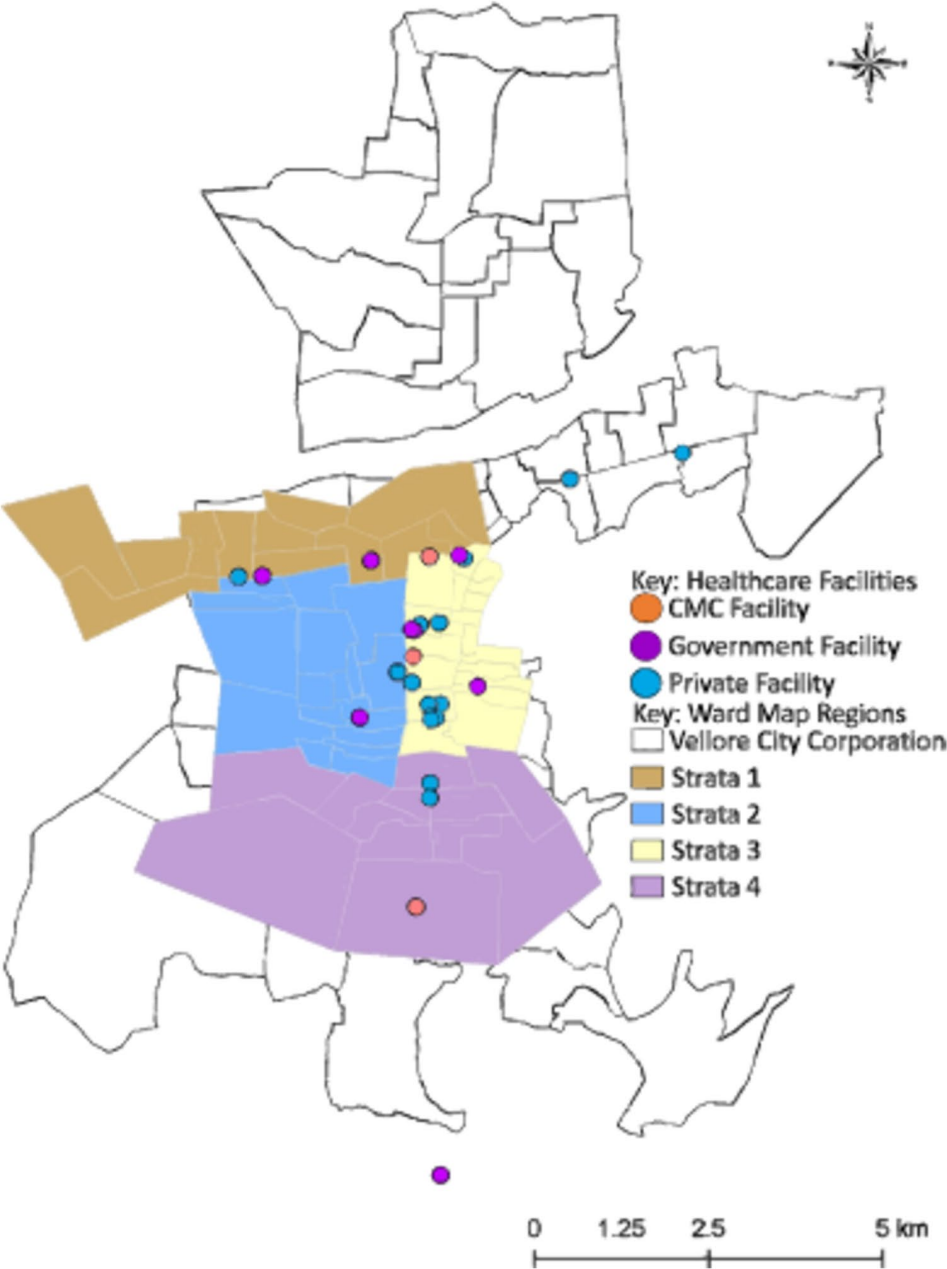
Ward map of Vellore city highlighting the trial clusters within the 4 randomisation strata

**Fig. 2:**
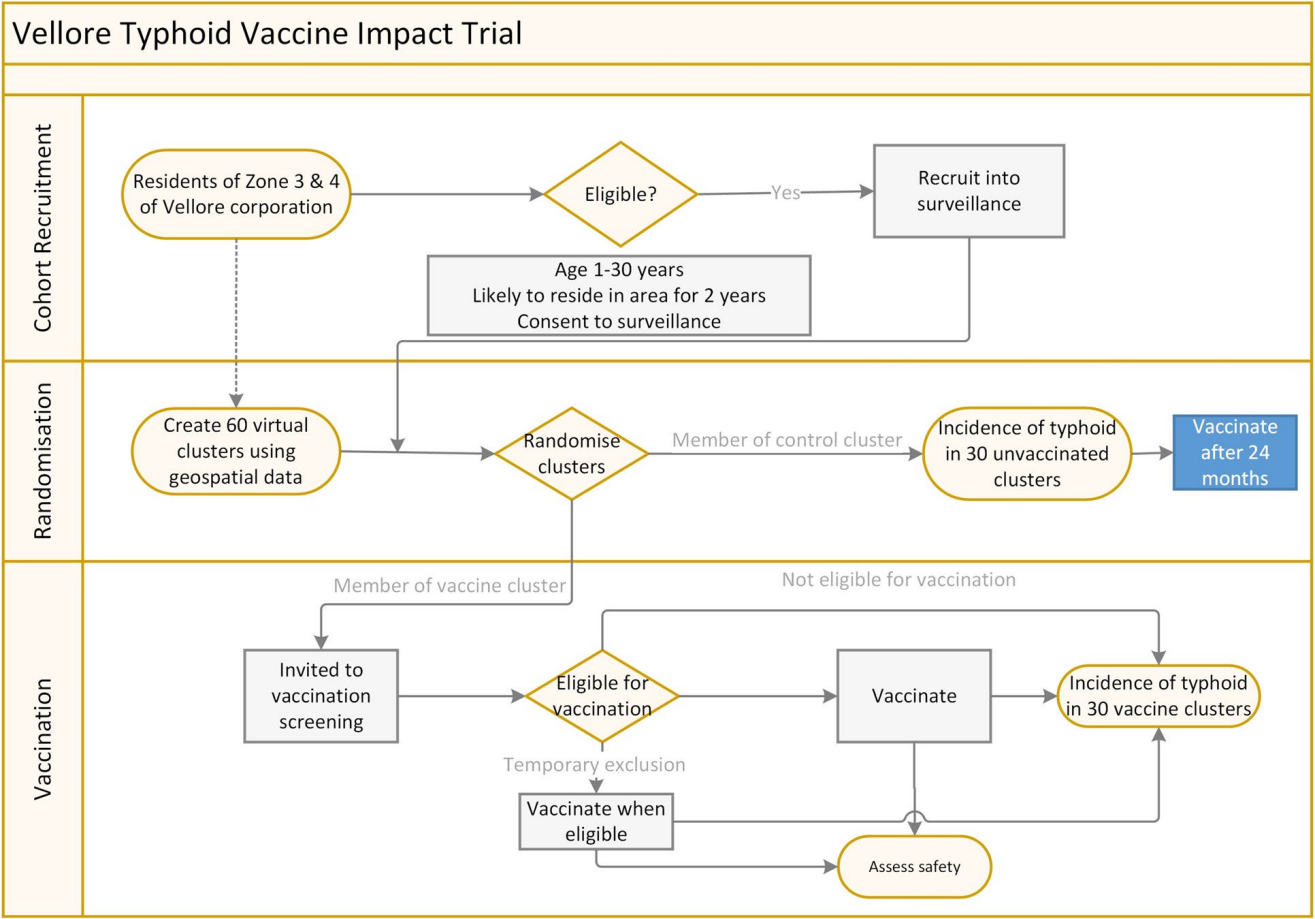
Flow diagram of the study processes

Our study is an observer-blinded, cluster-randomised trial set up among the population of 30 wards in Vellore that are part of the Vellore Health and Demographic Surveillance System (VDSS). Data from the VDSS will be updated with a door-to-door survey and individual residents between the ages of 1 and 30 years will be recruited into the study after informed consent. The wards will be divided into 60 geographically contiguous areas (Fig. 1), of which 30 will be randomly selected to receive TyphiBEV® (the remaining wards will receive this vaccine after the study follow-up period ends). Participants will be part of a stimulated passive surveillance for enteric fever for 25 months. We will partner with major hospitals and healthcare facilities in the area to capture such events. The trial is designed to detect superiority of the TyphiBEV® vaccine in reducing typhoid fever. A flowchart of the study is provided in Fig. 2.

### Comparator

No control vaccine will be used. All those in the no-intervention arm will receive the vaccine after the study ends. Potential comparator vaccines, i.e. Hepatitis A and Japanese B Encephalitis, were considered to have non-compelling risk-to-benefit ratios by the Christian Medical College (CMC) Vellore’s Institutional Review Board (IRB), since Hepatitis A seroprevalence is high among children in India [11] and Japanese encephalitis is non-endemic in Vellore [12].

## Methods and analysis

### Healthcare provider network

We invited the most utilised healthcare providers in the study area (determined by previous surveys) to participate in the fever surveillance based on their ability to follow a standard fever illness management protocol, i.e. to conduct a blood culture prior to the start of antimicrobial treatment in febrile patients. The locations of healthcare providers that have agreed to participate are highlighted in Fig. 1. This will include CMC Vellore and its subsidiary facilities, and other public and private healthcare centres.

### Eligibility and recruitment

Field Research Assistants (FRAs) will survey all households in the study area to identify individuals listed in the VDSS and those that may have migrated in or out. They will then approach individuals between the ages of 1 and 30 years for informed consent, and those who consent and do not plan to leave the study area within the next 2 years will be recruited. We will exclude those who have medical conditions that would prevent them from complying with our study’s requirements, which include follow-ups and investigations during illness. A monthly follow-up will be conducted for all participants to check for any changes in residence. A ‘mop-up’ recruitment drive 6 months after the initial process will recruit new migrants into the area and those that are newly age eligible.

FRAs will also collect baseline data from participants after enrolment, which includes socio-demographic details, typhoid vaccination history, healthcare facility preferences, etc.

### Fever surveillance

To capture febrile events, all participants will be asked to call a 24/7 helpline number before they visit a healthcare facility for an illness. In case the illness is febrile, our helpline operators will raise a unique digital token that the fever surveillance team will use (via an online dashboard) to track the patient. If the patient visits a facility partnered with this study, the team will contact the patient and study personnel at the facility to ensure that appropriate investigations are conducted, including automated blood cultures (CMC Vellore’s Microbiology laboratory will conduct this if the facility is unable to do so), and collect details and the outcomes of illness. We will also cover the patient’s cost of care and transportation to and from the healthcare facility. In case the patient visits a non-partnered hospital, the team will contact the patient directly to collect details of their illness and outcomes. This approach is likely to encourage reporting of febrile episodes by the participants as previous surveys have found that most febrile illnesses are treated at private facilities and paid for out of pocket.

Additionally, surveillance teams will be stationed at the major hospitals listed previously, to check for febrile in-patient (IP) cases at IP wards and emergency departments that may not have contacted the helpline or been missed by them. Further, their microbiology laboratory registers will be monitored for any enteric fever cases. At the end of each month, all participants will be contacted by the surveillance team to collect details about any illness episodes and remind them to contact the helpline prior to a healthcare facility visit.

This surveillance will end 25 months after vaccination and mark the trial’s end.

### Laboratory methods

All healthcare facilities partnered with the study will be encouraged to conduct a blood culture in case a patient has an acute febrile illness that lasts more than 2 days, has a temperature of ≥ 38 °C documented at the facility, or if the physician intends to begin antimicrobial therapy. The physician may defer the blood culture in case no antimicrobials are being initiated, if there is a confirmed alternate aetiology that excludes enteric fever, or if the patient has a documented negative blood culture report within the past 7 days.

All blood cultures will be collected under aseptic conditions and as per study SOPs. The blood volumes to be collected and inoculated into blood culture bottles (BacT/Alert) will be 5 mL for children between 1 and 15 years of age, and 8–10 mL for those ≥ 15 years. In case the study physician requires additional blood for investigations, it will be drawn during the same phlebotomy procedure. The weights of culture bottles before and after sample collection will be documented, along with the dates/times of loading into the BacT/Alert machine and of positivity. Any blood or bone-marrow cultures that are positive for *S.* Typhi will be considered as confirmed cases of typhoid fever. Those that are positive for either *S.* Typhi or *S.* Paratyphi will be considered as confirmed cases of enteric fever. These isolates will undergo antimicrobial susceptibility testing at the CMC Vellore Microbiology Laboratory, followed by genomic sequencing and archival as replicates in a − 80 °C storage facility.

Any additional serum samples collected from participants will also be stored at − 80 °C and tested for IgA HLyE antibodies, and other assays as indicated during their routine diagnosis.

### Vaccination

Once the fever surveillance is operational, eligible participants from selected vaccine clusters will be invited to designated vaccination clinics over a period of 2 months and have a unique vaccination code sent to their phones. This process will be repeated after the mop-up recruitment drive. In case of vaccination with any other vaccine within 30 days of the TyphiBEV® vaccination date, or a febrile illness in the week prior, the date will be rescheduled. Participants will be excluded if they decline consent, have received a typhoid vaccine in the past 3 years, are allergic to any component of TyphiBEV®, are nursing mothers, are pregnant, or are planning pregnancy around the vaccination date. If a participant is absent on their scheduled vaccination date, or subsequent rescheduled date, and does not respond to two reminders sent 7 days apart, they will be considered as having refused the vaccine. Participants that are ineligible or refuse the vaccine will continue to be part of the fever surveillance.

At the clinic, a trained vaccinator will check each participant’s unique code and government-issued identity card against the study database to verify their assignment to the intervention arm, and then dispense a single 0.5-mL, intramuscular dose of TyphiBEV® to eligible participants. Vaccinators, a doctor, and a nurse will make up the vaccination team at each clinic, which will also observe the patient for 30 min after vaccination in case of any immediate adverse events. The first 3600 vaccinated participants will be asked to maintain an adverse events diary for 7 days. Participants will also be contacted every month to check for any illnesses in that period.

Serious adverse events (SAEs) will be monitored by a study investigator who will inform the study’s Principal Investigator (PI) of them. Any that are unexpected and related to the vaccination in the PI’s opinion will be reported to the CMC IRB within 24 h of the PI being aware of them, using the SAE reporting form. Trial-related adverse events will be treated as per CMC Vellore’s institutional guidelines.

### Blinding

The study participants will not be blinded to their vaccination statuses as there is no control vaccine. Only the teams conducting fever surveillance and monthly contacts will be blinded to this and be trained to not seek out vaccination details. The coding for the primary analysis will be first conducted in a dataset with ‘dummy’ cluster and trial arm identifiers, and only then applied to the full dataset. This will be replicated by an independent analyst, who has not viewed the original results.

### Sample size calculation

The sample size was calculated using a simulation approach as outcome is an infectious disease that affects the probability of further infections in that cluster. The required number of participants was sampled from the VDSS 2021 dataset so that the simulation used the empirical distributions of age and household size in the study area (to explore potential sample sizes larger than the VDSS dataset, we replicated it before sampling). The ‘households’ were subsequently set within clusters of the required size, these clusters were randomised, and the discrete-time (one-month blocks) simulation began. We made conservative assumptions about the population proportion under 30 (47% assumed), typhoid incidence rate in the control arm (190 per 100,000 per year across all age groups combined), vaccine uptake (as low as 75%), and 70% reduction in risk due to vaccination. Further details of the simulations are provided in Additional file 2.

The simulation model reflected several important features of the trial but considered cluster membership to be constant throughout the 2 years of the trial and so did not represent the anticipated in- and out-migration.

Through this trial, we will test the null hypothesis of no vaccine effect on the incidence rate of blood culture-confirmed typhoid fever among participants. The sample size required to achieve this was determined to be 60 clusters of approximately 2800 individuals each. This will provide our trial with > 90% power for both total and overall vaccine effects. It will also provide some power to demonstrate the vaccine’s effectiveness above specific margins.

### Randomisation

Randomisation will be stratified according to four broad areas in the city with different anticipated incidences of *S.* Typhi (Fig. 1). The randomisation will be further restricted so that no cluster can be surrounded by contiguous clusters with the same vaccine allocation. This will be achieved by generating possible stratified randomisation sequences, rejecting sequences in which this condition is not fulfilled, and finally selecting at random one of the remaining sequences. To maintain allocation concealment, one statistician will randomise clusters to codes 0 and 1 to represent trial arms, and a second statistician will independently randomly allocate the codes to intervention and control. The randomisation process will be overseen by an unblinded statistician, who will not be involved in the primary analysis of data.

### Outcomes

The primary outcome is blood culture-confirmed typhoid fever.

The secondary effectiveness outcomes are:

• Febrile illness episodes lasting at least 3 days where healthcare is sought

• Hospitalised culture-confirmed *S*. Typhi infection

The safety outcomes, defined only in vaccine recipients, are:

- o Local induration at site of vaccination
- o Reported fever
- o Headache
- o Fatigue/tiredness
- o Anorexia
- o Abdominal pain
- o Serious adverse events
- o Solicited and unsolicited adverse events

The key process outcome is the proportion of participants age 1–29 in intervention clusters that are vaccinated, and will be reported in a CONSORT flow chart.

### Estimands

To provide full clarity for our first two objectives, the corresponding estimands are described in Table 1.

**Table 1:** Structured statement of estimands

	**Total effect**	**Overall effect**
Description	Effect of offering vaccination to clusters in 1–30-year-olds who would take it	Effect of offering vaccination to clusters in 1–30-year-olds
Target population (to whom the treatment effect applies)	Residents of Vellore aged 1–30 years who would take their assigned treatment	Residents of Vellore aged 1–30 years
Treatment condition (including the population exposed to an offer)	TyphiBEV® vaccination of those aged 1–30 years, including catch-up for late recruits (but not for participants who move from a control to a vaccination cluster)	Offer of TyphiBEV® vaccination to those aged 1–30 years, including catch-up for late recruits (but not for participants who move from a control to a vaccination cluster)
Endpoint	Blood culture-confirmed typhoid fever within 2 years	Blood culture-confirmed typhoid fever within 2 years
Summary measure/s	Marginal individual average incidence rate ratio	Marginal individual average incidence rate ratio
Intercurrent event handling	Refusal of vaccine offer by participants in vaccine clusters will be handled using a principal stratum strategy (which is our interpretation of the total effect), as will taking of vaccine by participants in non-vaccine clusters	Refusal of vaccine will be handled using a treatment policy strategy (which corresponds to the overall effect)
	**Migration**	**Migration**
	*Out of the study area* will be handled by censoring the participant at the point they leave	*Out of the study area* will be handled by censoring the participant at the point they leave
	*Into study area* will be treated as late recruitment	*Into study area* will be treated as late recruitment
	*From control cluster to control cluster* does not affect the estimand	*From control cluster to control cluster* does not affect the estimand
	*From vaccine cluster to vaccine cluster* does not affect the estimand	*From vaccine cluster to vaccine cluster* does not affect the estimand
	*From vaccine cluster to control cluster* will be censored	*From vaccine cluster to control cluster* will be censored
	*From control cluster to vaccine cluster* will be censored	*From control cluster to vaccine cluster* will be censored

### Statistical analysis

Our primary summary measure of the effect is a rate ratio (RR), from which vaccine effectiveness can be expressed as (1 − RR) × 100%. This will be estimated using an individual-level analysis using Poisson regression that will account for time at risk with censoring at the last known follow-up time. This is expected to be less than 2 years when participants migrate out of the study area. We will present an adjusted RR from a regression model including, covariates, the randomisation strata, participant age, gender, and household size. We will also compute unadjusted RRs but the adjusted RR will be considered ‘primary’. A difference in rates will also be computed after fitting the regression model, by standardization [13]. We will present both measures with 95% confidence intervals and two-sided hypothesis tests that consider a *p*-value of < 0.05 to be significant. Inference will be based on the bootstrap for the total effect and will use robust standard errors to account for clustering for the overall effect.

For measures of vaccine effectiveness which are described here as contrasts between vaccinated participants in vaccine clusters and unvaccinated participants in non-vaccine clusters, such as the total effect, we will use instrumental variable methods. Under this approach, while data from all participants are included in the analysis, we estimate the effect of vaccine only in those who would take it if available in their cluster (i.e. would ‘comply’). Although we do not observe this compliance in the non-vaccine arm, by randomisation we would expect the same proportion as in the vaccine arm, and this underlies the approach. The details of exactly how this will be done will be specified in subsequent versions of the statistical analysis plan.

The time at risk for the overall effect will begin 28 days after vaccination is completed in all intervention clusters. For participants that move into a trial cluster from outside the trial area, time at risk for the overall effect will begin at recruitment. For the total effect, time at risk for non-vaccine clusters begins as for the overall effect. In vaccine clusters, this will begin 28 days after vaccination is completed across clusters or 28 days after an individual is vaccinated (whichever is later) and no risk-time is contributed by those unvaccinated. Time at risk ends at the study’s close, the time of an event (culture-confirmed typhoid fever), withdrawal from the study, or migration from the study area or within the area but between trial arms (whichever is earliest).

The primary outcome might have missing data if a trial participant with a febrile illness visits a study-associated healthcare facility but refuses a blood culture. We will describe the frequency of such refusals by trial arm. We will multiply impute the blood culture results for these participants under the missing at random assumption and conduct sensitivity analyses to explore the impact on the total and overall vaccine effect estimates of making alternative assumptions.

We do not have any plans for interim analyses of vaccine effectiveness, or subgroup analyses except that we will estimate the total effect in participants aged 1–14 years. We will conduct simple descriptive analyses of safety events in vaccinated participants, and of vaccine uptake in eligible vaccine arm participants.

A first draft of a detailed statistical analysis plan has been created and will be finalised before analysis.

### Data management, confidentiality, and quality assurance (QA)

Operational management of the trial will be aided by an externally validated, custom-built database management software. It will link with the VDSS and be used for recruitment, scheduling, and tracking of participant contacts and outcomes. Primary clinical data will be recorded on validated instances of Redcap (Research Electronic Data Capture) [14, 15] software forms, hosted on hybrid cloud servers within India, with built-in range and value checks. A dedicated data management team will generate regular data queries and backups. Access to the custom software and Redcap forms will be password protected and conducted via pre-approved tablets or laptop computers. Activities such as data entry, backup, validation, and export will be conducted as per SOPs and appropriately logged.

We will consider any document where demographic, clinical, or laboratory data is first recorded prior to entry into our digital Case Record Forms (CRFs) as source data. This includes hospital records, clinical charts, office charts, pharmacy, and laboratory records. If hospital data is unavailable in electronic format, the study team will maintain scanned copies until the CRFs are verified. If a CRF is the first place where data is entered, it will be considered source data. Hardcopy study documents will be stored securely in a confidential location. To maintain the anonymity of participants, they will only be referred to by their participant ID on study documents, except in informed consent sheets. All data collected during the trial will be stored at the site for at least 3 years after its completion. The PI will have complete access to the final dataset.

A dedicated QA team will regularly validate a proportion of the data entered into CRFs and ensure that all trial processes follow the protocol and Good Clinical Practices. The microbiology labs that process blood samples will participate in an external QA programme. The study processes will be reviewed by an external monitoring team at the end of one year.

### Trial oversight

A Trial Steering Group (TSG), comprised of the lead investigators, the data manager, internal quality manager, and the trial statisticians, will meet at least once a month to discuss trial operations. Interim reports will be prepared for the independent Data Safety and Monitoring Board (DSMB), comprised of an epidemiologist, statistician, paediatrician, physician, and a clinical pharmacologist, to address the recruitment of participants, vaccine uptake, and event rates in both arms of the trial. The DSMB will review these data to decide whether any additional sample size calculations are required, and whether to recommend an extension of the trial to allow more outcome events to accumulate. The board will advise the TSG on changes that may be required in the trial’s operations to achieve its objectives.

A Scientific Advisory Group, comprised of the lead investigators, independent experts in clinical trials and community interventions, representatives from the Directorate of Public Health, and policy experts, will also review the processes and progress of the trial once every 6 months and provide feedback.

## Discussion

TCVs are highly efficacious interventions against typhoid that are cost-effective and can be used to protect children from the age of 6 months [8]. This has been demonstrated via the use of Typbar TCV, manufactured by Bharat Biotech in India, for typhoid control in high-burden countries such as Pakistan and Zimbabwe [6]. As countries begin to introduce TCVs, having multiple manufacturers can help guard against vaccine supply constraints and promote competitive pricing. The TyphiBEV® vaccine trial will generate efficacy data and examine the impact of TCVs in a high-burden setting.

Efficacy studies of TCVs in various settings, regions, and age groups are necessary to help with their effective programmatic use. Our trial’s results will also inform the Indian Government on the effectiveness of TyphiBEV® among children and young adults in urban settings, which they may use for a country-wide rollout of TCVs.

## Trial status

**Fig. 3:**
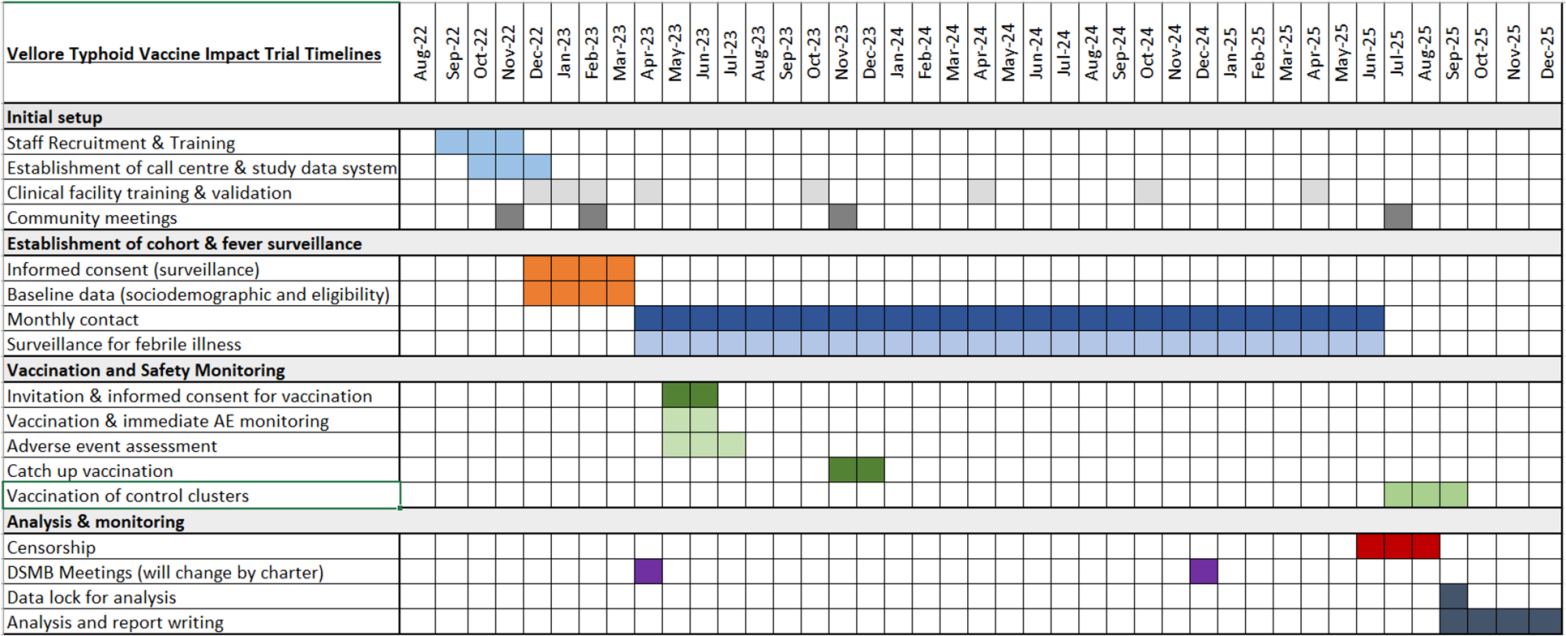
Timeline of study procedures (SPIRIT figure)

This manuscript is based on protocol version 1.1, dated 26/04/2023 and titled ‘Vellore Typhoid Vaccine Impact Trial’. A timeline of the study can be found in Fig. 3. Recruitment of trial participants started on 28/11/2022 and was completed by 29/03/2023, but randomisation of clusters has not occurred at the time of submission.

## Supplementary Information

**Additional file 1.** SPIRIT Checklist.

**Additional file 2.** Simulation methodology and results for power and sample size.

## Data Availability

De-identified, participant-level data will be made available via a public archive within 3 months of the primary publication, along with the statistical analysis plan and analysis code. Genome sequences obtained will be uploaded to a sequence library. Supplementary analyses may be conducted on this data with attribution to the source. The template consent and participant information sheets and data collection forms can be obtained from the corresponding author on request.

## Abbreviations

CMC: Christian Medical College
CRF: Case Record Form
CTRI: Clinical Trials Registry of India
DSMB: Data Safety and Monitoring Board
FRA: Field Research Assistant
IP: In-patient
IRB: Institutional Review Board
PI: Principal Investigator
QA: Quality assurance
Redcap: Research Electronic Data Capture
RR: Rate ratio
SAE: Serious adverse event
SEFI: Surveillance for Enteric Fever in India
TCV: Typhoid conjugate vaccine
TSG: Trial Steering Group
VDSS: Vellore Health and Demographic Surveillance System
WHO: World Health Organization

## References

[1] The Institute for Health Metrics and Evaluation. https://www.healthdata.org/results/gbd_summaries/2019/typhoid-and-paratyphoid-level-3-cause. Accessed 3 Feb 2023.

[2] Stanaway JD, Reiner RC, Blacker BF, Goldberg EM, Khalil IA, Troeger CE (2019). The global burden of typhoid and paratyphoid fevers: a systematic analysis for the Global Burden of Disease Study 2017. Lancet Infect Dis.

[3] Garrett DO, Longley AT, Aiemjoy K, Yousafzai MT, Hemlock C, Yu AT (2022). Incidence of typhoid and paratyphoid fever in Bangladesh, Nepal, and Pakistan: results of the Surveillance for Enteric Fever in Asia Project. Lancet Glob Heal.

[4] John J, Bavdekar A, Rongsen-Chandola T, Dutta S, Gupta M, Kanungo S (2023). Burden of typhoid and paratyphoid fever in India. N Engl J Med.

[5] Butt MH, Saleem A, Javed SO, Ullah I, Rehman MU, Islam N (2021). Rising XDR-typhoid fever cases in Pakistan: are we heading back to the pre-antibiotic era?. Front Public Heal.

[6] Birkhold M, Mwisongo A, Pollard AJ, Neuzil KM (2021). Typhoid conjugate vaccines: advancing the research and public health agendas. J Infect Dis.

[7] Thuluva S, Paradkar V, Matur R, Turaga K, Gv SR (2022). A multicenter, single-blind, randomized, phase-2/3 study to evaluate immunogenicity and safety of a single intramuscular dose of biological E’s Vi-capsular polysaccharide-CRM197 conjugate typhoid vaccine (TyphiBEVTM) in healthy infants, children, and adults in comparison with a licensed comparator. Hum Vaccin Immunother.

[8] Ryckman T, Karthikeyan AS, Kumar D, Cao Y, Kang G, Goldhaber-Fiebert JD (2021). Comparison of strategies for typhoid conjugate vaccine introduction in India: a cost-effectiveness modeling study. J Infect Dis.

[9] Chauhan AS, Kapoor I, Rana SK, Kumar D, Gupta M, John J (2021). Cost effectiveness of typhoid vaccination in India. Vaccine.

[10] Chan AW, Tetzlaff JM, Gøtzsche PC, Altman DG, Mann H, Berlin JA, SPIRIT,  (2013). explanation and elaboration: guidance for protocols of clinical trials. BMJ.

[11] Murhekar MV, Ashok M, Kanagasabai K, Joshua V, Ravi M, Sabarinathan R (2018). Epidemiology of hepatitis A and hepatitis E based on laboratory surveillance data—India, 2014–2017. Am J Trop Med Hyg.

[12] Government of Tamil Nadu Health and Family Welfare Department. https://tnhealth.tn.gov.in/tngovin/dph/dphdbje.php. Accessed 21 Mar 2023.

[13] Morris TP, Walker AS, Williamson EJ, White IR (2022). Planning a method for covariate adjustment in individually randomised trials: a practical guide. Trials.

[14] Harris PA, Taylor R, Minor BL, Elliott V, Fernandez M, O’Neal L (2019). The REDCap consortium: building an international community of software platform partners. J Biomed Inform.

[15] Harris PA, Taylor R, Thielke R, Payne J, Gonzalez N, Conde JG (2009). Research electronic data capture (REDCap)—a metadata-driven methodology and workflow process for providing translational research informatics support. J Biomed Inform.

