# Supplementary material for: An observer-blinded, cluster randomised trial of a typhoid conjugate vaccine in an urban South Indian cohort
Source: Trials. 2023 Aug 3;24:492. doi: 10.1186/s13063-023-07555-y (PMC10399005; doi:10.1186/s13063-023-07555-y)
Supplement: Supplementary file 2 — Additional file 2. Simulation methodology and results for power and sample size. [file 13063_2023_7555_MOESM2_ESM.pdf]

# 1. ADDITIONAL FILE – SIMULATION METHODOLOGY AND RESULTS FOR POWER AND SAMPLE SIZE

## 1.1. Aim

The aim is to use simulation of hypothetical data to explore and inform the possible design of a cluster-randomised trial to evaluate typhoid vaccine. This is necessary for various reasons. Typhoid is infectious and so observed outcomes within a cluster are not independent (we anticipate some indirect effect of a cluster vaccine policy). We have some empirical data on the age and household distributions, which are important in this setting and can be readily included in simulations. We also have empirical data concerning the variability between clusters, and we cross check ('calibrate') our simulations to match this variability (looking at the distribution of cluster summary incidence values, rather than simply summarising the variability by a single number such as the intra-cluster coefficient). Uptake of vaccination among those eligible will not be perfect and we can explore the impact of this.

The design of a cluster-randomised trial will be such that clusters are not pre-existing but are defined for the trial, meaning we are able to control cluster size and make this approximately equal. Our original aims were to explore:

How many clusters to include in total (looking in the range of about 50 to 120).
How many people per cluster.
If it would be feasible to run either (a) a three-arm trial that includes vaccination of under-45y alongside a control arm and a vaccination of under-30y arm, or (b) a two-arm trial of control and vaccination under-30y, but with data collection up to age 44 to allow indirect effects on those aged 30-44 to be explored.
In this document we focus on the final trial design as reflected in the rest of the SAP (two arm trial, outcome data collected only from those aged 1-29, 60 clusters, 2800 population each) but include calculations for the alternative design where data are collected from those aged 1-44 so as to illustrate and explain the choice made.

## 1.2. Simulation outline

- **Data-generating mechanism**

The setup and logic of the simulation is outlined below and Stata code is available for details.

We start by reading in the DSS data, which gives us the empirical distribution of household size and individuals' ages. This is important because age is prognostic for a typhoid infection and infection within a household will increase the probability of other individuals within that household being infected.

The data are then split into clusters of approximately equal size and the clusters are assigned random intercepts by drawing from a (scaled) distribution. These draws will represent the cluster's point-prevalence of typhoid at time 0. The specific distribution was chosen to return similar between-cluster variability seen in empirical data if clusters

were the same size as in the empirical data. This point is important because as the cluster size increases the variance reduces. This returns a distribution where most clusters have low incidence and a minority will experience higher incidence (small outbreaks).

Clusters are block-randomised so that half are assigned to each arm (exactly half, since we only consider even numbers of clusters). In the simulation, randomisation is unstratified, though in the trial itself randomisation of clusters will be stratified, giving the study slightly higher power.

A small proportion of individuals are assigned asymptomatic ‘potential carrier’ status. Some are carriers at time 0 but some only become carriers if infected during the trial. They then remain infectious to others for the duration of the trial.

Typhoid infections as time 0 are then simulated from a binomial distribution conditional on predicted probability of infection and the time-dynamic part of the simulation begins.

The uptake of vaccination among those eligible is less than 100% and we 85% as our baseline but also explore 75%.

We take a discrete-time approach to simulating infections over two years (the proposed length of the trial). Because a typhoid infection involves an initial infectious period [around 2 days?] followed by a less infectious period [3 to 14 days] followed by another infectious period [~15 to 28 days], we use discrete time blocks of four weeks. Twenty-six of these blocks make up the two-year period.

At each time-point, a simulated individual’s probability of infection without vaccination depends on age, whether any individual within their household had an infection at time , the level of infection within their cluster at time  and the proportion of infections across all clusters at time  (to represent the fact that individuals move between the clusters in which they live and, for the purposes of the trial, to which they are assumed to ‘belong’), so that a high incidence in one cluster is subsequently likely to affect other clusters. This results in contamination across clusters, though in the trial we would make efforts to leave space between clusters and minimise the effects of this contamination. The intercept of the model that updates infection status was calibrated so that, with no vaccination, we would expect approximately 190 cases per 100,000 annually.

Among those randomised to vaccination, receipt of the vaccine leads to a 70% reduction in the relative risk of infection under the alternative hypothesis. Under the null, receipt of the vaccine does not change probability of infection.

Among those with a typhoid infection, asymptomatic carriers are assumed to have a continued infection for the duration of the trial. Everyone else’s infection is assumed to last for under 4 weeks and, following this, they are immune and not susceptible.

The outcome measure is any infection during the trial. This is summarised for each individual.

- **Estimands**

  We explore four estimands of interest. For the purpose of these simulations, the summary measure is the risk ratio, noting that in the trial the absolute risk difference would also be summarised.

  In the below, we distinguish between the effect type in terms of people who would or would not be eligible for vaccination under any policy, and then the strategy for handling non-receipt among those who would be eligible. Note that this terminology may differ slightly from that used in the literature because the policy considered involves vaccination depending on age. This is important because, for example, individuals over 30 would not be eligible for vaccination but may benefit indirectly from vaccination of the younger, higher-risk individuals within their cluster.

  - **Overall effect in 0–45y** estimated using a **treatment policy strategy** for handling non-receipt of vaccination. This is the effect on individuals under 45y of the policy to offer vaccination to those under 30y.
  - **Overall effect in 0–30y** estimated using a **treatment policy strategy** for handling non-receipt of vaccination. This includes individuals under 30y only as those who would have been eligible for vaccination under this policy, but with interest in the effect of the offer to this high-risk population. This is the effect on individuals under 30y of the policy to offer vaccination to those under 30y.
  - **Total effect in 0–30y** estimated using a **principal stratum strategy** for handling non-receipt of vaccination. This includes individuals under 30y only as those who would have been eligible for vaccination under this policy, but with interest in the effect of receiving vaccination on this higher-risk population. This is the effect on individuals under 30y of receiving vaccination those under 30y supposing everyone had received it.
  - **Indirect effect in 30–45y**, where there is no intercurrent event since this group was not offered treatment. This includes individuals of 30–45y. It is an indirect effect of the benefit of being in a vaccinated cluster where vaccinated individuals may confer protection on non-vaccinated.

- **Methods of analysis**

  Initial exploration of a cluster-summaries analysis found issues including large bias and poor control of type I error rate, likely due to being at the edge of the parameter space, so our analysis method is individual level.

  Individual-level data are analysed using a Poisson model for (binary) *confirmed S. Typhi infection during trial,* adjusted for the covariates *age* and *baseline cluster infection rate*. A Poisson model was chosen due in part to the canonical link function, which ensures good probability of convergence, convergence to a unique maximum, and robustness to model misspecification. Cluster-robust variance estimation is used. The individuals included in the analysis to estimate the estimand of interest are as follows:

  - **Overall effect** (1-44) with a **treatment policy strategy** includes all individuals under 45y and contrast is in terms of randomisation (i.e. the ITT population)
  - **Overall effect** (1-29) with a **treatment policy strategy** includes all individuals under 30y and the contrast is in terms of randomisation (i.e. the ITT population among under-30s)

- **Total effect** with a **hypothetical strategy** includes all individuals under 30y in the control arm and individuals under 30y who were vaccinated in the vaccination arm
- **Indirect effect** includes all individuals aged 30–45 and compares randomised arms.

Note that, although analyses are restricted to individuals under a defined age, older people are still represented in the generated data. This is important for example for asymptomatic carriers, who are likely to be older people with gall bladder issues.

- **Performance measures**

Primary interest is in power and type I error rate. Type I error rate is of interest because cluster-robust variance estimation is based on asymptotic arguments. Bias is hard to evaluate because we do not have a 'true' value for any of the above estimands due to the complexity of the data-generating model.

### 1.3. Results for final design configurations considered

An important point to note is that the input vaccine efficacy does not correspond to the cluster-level effect used as input: the cluster-level risk ratio is more modest than the input value of 0.25.

After exploration of various designs, in particular difference cluster size and number of clusters, we settled on 60 clusters with a total cluster size of 2,800 (all individuals; with 47% expected to be under 30 years at baseline).

- **Type I Error**

The table gives the type I error proportion for various estimands.

| Effect | Age group | Type I error (MCSE) |
|---|---|---|
| Indirect | 30–45y | 7.4% (0.83) |
| Overall | 0–45y | 9.0% (0.90) |
| Overall | 0–30y | 8.8% (0.90) |
| Total | 0–30y | 8.0% (0.86) |

We see that type I error is higher than the nominal 5% for all scenarios and this does not appear to be compatible with Monte Carlo error. This is not ideal for two reasons:

- We would want to have type I error rate controlled at <=5%. Power results (below) appear slightly more favorable than they would be with type I error rate of exactly 5%.
- We believe this occurs due to unmodelled dependence within clusters. For the analysis of the trial we will look into alternative methods for constructing tests and confidence intervals based on bootstrapping, and run further simulation studies to evaluate their properties.

- **Power**

Results for power are given in the following table. The first column is for superiority, with subsequent columns giving power for super-superiority with various margins. The cells in bold show power for the primary estimand of interest, assuming 70% vaccine coverage in vaccine cluster. To demonstrate superiority, power is extremely high. Power is also high for super-superiority, at least 83% to demonstrate effectiveness greater than 20% (I.e. null RR=0.8).

| Vaccine coverage | Effect | Age group | Null = 1 | Null=0.9 | Null=0.85 | Null=0.8 |
|---|---|---|---|---|---|---|
| 70% | Indirect | 30–45y | 14% | 5% | 3% | 1% |
| | Overall | 0–45y | 96% | 83% | 72% | 54% |
| | Overall | 0–30y | **99%** | **95%** | **91%** | **83%** |
| | Total | 0–30y | **100%** | **100%** | **99%** | **98%** |
| 80% | Indirect | 30–45y | 16% | 8% | 5% | 3% |
| | Overall | 0–45y | 98% | 94% | 88% | 77% |
| | Overall | 0–30y | 100% | 99% | 98% | 94% |
| | Total | 0–30y | 100% | 100% | 100% | 99% |
